# Genome-Wide Methylated DNA Immunoprecipitation Analysis of Patients with Polycystic Ovary Syndrome

**DOI:** 10.1371/journal.pone.0064801

**Published:** 2013-05-21

**Authors:** Hao-ran Shen, Li-hua Qiu, Zhi-qing Zhang, Yuan-yuan Qin, Cong Cao, Wen Di

**Affiliations:** 1 Department of Obstetrics and Gynecology, Ren Ji Hospital, School of Medicine, Shanghai Jiao Tong University, Shanghai, China; 2 Institute of Neuroscience, Soochow University, Suzhou, Jiangsu, China; University of Warwick – Medical School, United Kingdom

## Abstract

Polycystic ovary syndrome (PCOS) is a complex, heterogeneous disorder of uncertain etiology. Recent studies suggested that insulin resistance (IR) plays an important role in the development of PCOS. In the current study, we aimed to investigate the molecular mechanism of IR in PCOS. We employed genome-wide methylated DNA immunoprecipitation (MeDIP) analysis to characterize genes that are differentially methylated in PCOS patients vs. healthy controls. Besides, we also identified the differentially methylated genes between patients with PCOS-non-insulin resistance (PCOS-NIR) and PCOS-insulin resistance (PCOS-IR). A total of 79 genes were differentially methylated between PCOS-NIR vs. PCOS-IR patients, and 40 genes were differentially methylated in PCOS patients vs. healthy controls. We analyzed these differentially methylated genes by constructing regulatory networks and protein-protein interaction (PPI) networks. Further, Gene Ontology (GO) and pathway enrichment analysis were also performed to investigate the biological functions of networks. We identified multiple categories of genes that were differentially methylated between PCOS-NIR and PCOS-IR patients, or between PCOS patients and healthy controls. Significantly, GO categories of immune response were differentially methylated in PCOS-IR vs. PCOS-NIR. Further, genes in cancer pathways were also differentially methylated in PCOS-NIR vs. PCOS-IR patients or in PCOS patients vs. healthy controls. The results of this current study will help to further understand the mechanism of PCOS.

## Introduction

Polycystic ovary syndrome (PCOS) is a complex, heterogeneous disorder of uncertain etiology. Strong evidence suggest that it can be classified as a genetic [1], [2], [3] and epigenetic disorders [4]. Such evidence include the familial clustering of cases, greater concordance in monozygotic compared with dizygotic twins and heritability of endocrine and metabolic features of PCOS [5]. PCOS is one of the leading causes of female subfertility and is seen in approximately 5%–10% of women of 12–45 years old [6], [7], [8].

The features of PCOS include chronic anovulation or few ovulations, polycystic ovaries enlargement and hyperandrogenism. In addition, PCOS patients are often accompanied with insulin resistance (IR) and β-cell dysfunction [9]. Further, patients with PCOS have decreased conception rate, and increased prevalence rates of spontaneous abortion and gestational diabetes [10], [11]. Besides, PCOS patients are at higher risk of suffering from endometrial carcinoma. Recent studies show that PCOS patient's incidence of metabolic syndrome (MS) is also higher [12], which is associated with cardiovascular diseases and IR. At present, many scholars have been focusing on the relationship between IR and PCOS, and results show that PCOS patients' endocrine condition and their reproduction can be relieved by ameliorating their IR. Life style adjustment can be an efficient way to achieve this goal. Besides, oral hypoglycemic agents are also subscribed to treat the IR in PCOS patients [13].

Recent studies have elaborated that inappropriate epigenetic reprogramming is an important contributing factor for PCOS [14], [15], [16]. However, the concrete mechanisms of epigenetic alterations and downstream signal cross-talk responsible for PCOS are remaining largely unknown. We employed genome-wide methylated DNA immunoprecipitation (MeDIP) analysis to characterize methylated genes in patients with PCOS vs. healthy controls. Besides, we also identified the differentially methylated genes between patients with PCOS-non-insulin resistance (PCOS-NIR) and PCOS-insulin resistance (PCOS-IR).

## Materials and Methods

### Sample collection

The study was approved by the institutional review board of Renji Hospital, Shanghai Jiao Tong University School of Medicine, and written informed consent was obtained from all patients. All clinical investigations were conducted according to the principles expressed in the Declaration of Helsinki. Our subjects included 10 unrelated female patients with PCOS (5 PCOS-NIR patients and 5 PCOS-IR patients) and 5 unrelated female healthy controls. These subjects were selected from an existing cohort of 86 cases and 44 controls, which were recruited at Renji Hospital affiliated to Shanghai Jiao Tong University School of Medicine. PCOS was defined according to criteria of the Androgen Excess Society (AES) at 2006 [17]. All cases and controls in this study did not take hormone therapy for at least 3 months. Serum total testosterone (TT) and fasting insulin (FINS) were assayed by radioimmunoassay (RIA) (Beckman Coulter, Inc. Shanghai, China). Serum free testosterone (FT) and sex hormone binding globulin (SHBG) were determined by RIA kit (Beckman Coulter, Inc. California, USA) according to the manufacturer's instructions. Serum fasting blood-glucose (FBG) was determined by the glucose oxidase method (Sysmex Corporation, Shanghai, China). Typical values for the free androgen index (FAI; calculated by the equation FAI = TT×100/SHBG) in women were 7–10 [18]. Homeostatic model assessment IR (HOMA-IR; calculated by the equation HOMA-IR = FBG×FINS/22.5)≥2.5 indicates IR [19]. Peripheral blood samples were extracted from all subjects for MeDIP analysis.

### Genome-wide methylated DNA immunoprecipitation (MeDIP) analysis

PCOS-associated and PCOS-IR-associated methylation profiles were gained from the MeDIP-chip platform (Shanghai Biochip, Shanghai, China) based on Nimblegen Human Meth 3×720K CpGRfSq Prom Arr Del (Roche NimbleGen, Wisconsin, USA). Each subject's sample was analyzed with one MeDIP-chip separately. Genomic DNA extracted from peripheral blood sample of the 5 controls and 10 PCOS patients (5 PCOS-NIR patients and 5 PCOS-IR patients) was prepared using the DNeasy Blood & Tissue kits (Qiagen, USA). About 2 µg of DNA was bisulfite-treated with the EpiTect Bisufite kit (Qiagen, USA) following the manufacturer's protocol. Amplification across the entire bisulfate converted genome was performed by the EpiTect Whole Bisufitome kit (Qiagen, USA) according to the manufacture's protocol.

To verify the specificity of DNA methylation, we performed methylation-specific PCR (MSP). According to the principle of methylation, we designed the methylation-specific PCR primers for estrogen receptor beta (ER-β) by using MethPrimer (http://www.urogene.org/methprimer/), which were shown in Table 1. Genetic DNA extracted from peripheral blood sample of normal control, PCOS-NIR patients and PCOS-IR patients was amplified using methylated-specific primer (M) and unmethylated-specific primer (U). Positive control of methylation was achieved by using the EpiTect MSP kit (Qiagen, USA). Negative control of methylation was achieved by using distilled water.

**Table 1:** The primers for ER-β in this experiment.

Primer	Direction	Sequences (5′-3′)	Product size
Methylated-specific primer (M)	Forward	CGAGGGTGTTTTTATTTAGAGGTTAC	256 bp
	Reverse	ATTTCAAAAAACAATTATTTCTCGC	
Unmethylated-specific primer (U)	Forward	TGAGGGTGTTTTTATTTAGAGGTTAT	255 bp
	Reverse	TTTCAAAAAACAATTATTTCTCACA	

Before carrying out MeDIP, we sonicated genomic DNA to produce random fragments ranging in size from 300 bp to 1000 bp. MeDIP assay was carried out as described previously [20]. Briefly, the samples were independently labeled with Cy5 (IP) and Cy3 (INPUT) using a NimbleGen Dual Color DNA labeling kit (Roche NimbleGen, Wisconsin, USA). Co-hybridizations in dye-swap were performed using a NimbleGen Human Meth 3× 720K CpG RfSq Prom Arr Del array. After heat denaturation at 95°C for 10 min, DNA was incubated with antibody against 5- methylcytidine (Diagnode, Belgium) in 1× IP buffer (10 nM sodium phosphate, pH 7.0, 140 mM NaCl, 0.05% (w/v) Triton X-100) at 4°C overnight. Immune complex were collected with Dynabeads Protein A (Invitrogen, USA), washed with 1× IP buffer for seven times, treated with Proteinase K for 4 hours at 42°C, and purified by phenol and chloroform extraction and isopropanol precipitation. Then they were scanned using an AXON GenePix 4000B Microarray Scanner (AXON, California, USA).

Signals were localized and expression ratio between experimental and reference (Cy5/Cy3 ratio) was determined using by Nimblescan software V2.5 (Roche NimbleGen, Wisconsin, USA). The ratio was then log 2 transformed. Then the probability of genes (*p* value) being differentially methylated among groups was computed using ACME (Algorithm for Capturing Microarray Enrichment). The lower p value, the higher probability of probes being differentially methylated. Finally, peak score was calculated according to the p value of each probes (peak score = −lg P). The peak score indicates the reliability of peak. The probes with peak score >2 and p value<0.0005 may be the methylated regions.

### Transcription regulatory data

A total of 774 regulatory relations between 219 transcriptional factors and 265 target genes were collected from TRANSFAC (http://www.gene-regulation.com/pub/databases.html) and 5,722 regulatory relations between 102 transcriptional factors and 2, 920 target genes were collected from TRED (http://rulai.cshl.edu/TRED/). We integrated both groups and obtained 6,328 regulatory relations between 276 transcriptional factors and 3, 002 target genes. Based on these data, we constructed the regulatory network of PCOS-NIR/PCOS-IR, PCOS/healthy controls.

### Protein-protein interaction (PPI) network data

We collected 39, 240 PPIs from HPRD database [21] and 379, 426 protein-protein relations from BIOGRID database [22]. After integration for both databases, a total of 326,119 PPIs were obtained. Then, we mapped all the differentially methylated genes to the PPIs, and only kept the interactive differentially methylated genes and their nearest neighbor genes. Based on them, we constructed the PPIs network for PCOS-NIR/PCOS-IR, PCOS/healthy controls.

### Gene Ontology (GO) function and pathways analysis

The Database for Annotation, Visualization and Integrated Discovery [23] (DAVID) version 6.7 provides a comprehensive set of functional annotation tools to understand biological meaning behind large lists of genes. In our study, we used DAVID software (http://david.abcc.ncifcrf.gov/) to perform GO and PATHWAY analysis for regulatory network and PPI network.

### Statistical analyses

Data were analyzed with the IBM SPSS Statistics software V19.0 (IBM, New York, USA). Independent t tests were performed to evaluate the significance of any differences between test and control groups. All p-values were 2-sided, and *p*<0.05 was considered to be significant.

## Results

Clinical data were summarized in Table 2. The three groups were comparable in terms of age, height, weight, BMI, hormone and glucose levels. Serum total testosterone (TT), free testosterone (FT) and follicle count of PCOS patients were higher than healthy controls (*p*<0.05). The sex hormone binding globulin (SHBG) level in PCOS patients was lower compared with healthy controls (*p*<0.05). The levels of fasting insulin (FINS) and homeostatic model assessment insulin resistance (HOMA-IR) were higher in PCOS-IR patients than PCOS-NIR patients or healthy controls (*p*<0.05).

**Table 2:** baseline patients' characteristics.

	PCOS-NIR (n = 5)	PCOS-IR (n = 5)	Control (n = 5)
**Age (year)**	24.8±2.17	27.4±3.44	25.4±2.51
**Height (cm)**	162.6±3.44	158.4±3.21	161.4±3.13
**Weight (kg)**	56.2±3.90	66.6±1.52	51.2±6.30
**BMI (kg/m^2^)**	21.3±1.11	26.6±0.77	19.7±2.41
**TT (nmol/l)**	3.59±0.58	3.25±0.51	1.67±0.52
**SHBG (nmol/l)**	30.3±6.02	18.7±7.99	63.1±19.8
**FT (pg/ml)**	9.43±1.08	11.2±1.72	5.16±0.42
**FBG (mmol/l)**	5.07±0.32	5.42±0.36	5.24±0.27
**FINS (µIU/ml)**	8.42±3.08	13.4±3.51	6.54±0.79
**FAI**	12.0±1.56	19.8±7.55	2.87±1.34
**HOMA-IR**	1.87±0.55	3.18±0.67	1.53±0.22
**Follicle count**	15.6±1.34	14.4±2.30	6.2±1.30

Data are presented as means±standard deviations. PCOS, Polycystic ovary syndrome; IR, insulin resistance; NIR, non-insulin resistance; BMI, body mass index; TT, total testosterone; SHBG, sex hormone binding globulin; FT, free testosterone; FINS, fasting insulin; FBG, fasting blood glucose; HOMA-IR, homeostatic model assessment-insulin resistance = (FBG×FINS)/22.5; FAI, free androgen index = (TT×100)/SHBG.

### Specificity analysis of methylated DNA

**Figure 1:**
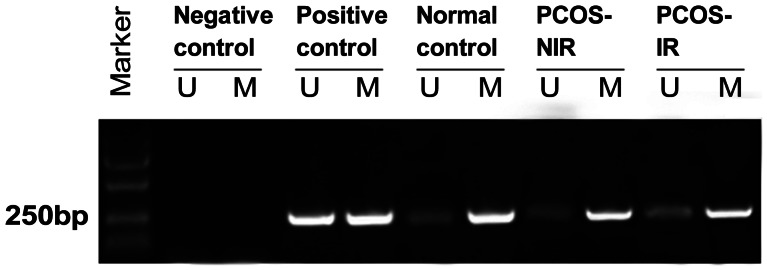
MS-PCR electrophoretogram showing the specificity of methylated DNA. Bands in the M lanes represent the methylated PCR products of ER-β, whereas bands in the U lanes represent the unmethylated ER-β. The presence of a 250 bp band indicates hypermethylated DNA fragment and the fuzzy band in the U lane indicates partially methylated DNA fragment.

To investigate the specificity of methylated DNA, MSP was performed. Genetic DNA was extracted from peripheral blood sample of normal control, PCOS-NIR and PCOS-IR patients, respectively. As shown in Figure 1, the fragment of approximate 250 bp was specifically appeared in samples amplified by M primer.

### Genome-wide methylated DNA immunoprecipitation (MeDIP) analysis identification of differentially methylated genes

We applied PCOS related and PCOS-IR related methylation profiles from MeDIP-chip platform. Fold-change and t-test methods were used to identify differentially methylated genes. Of the genes examined, 79 genes of them were identified as differentially methylated in PCOS-NIR patients vs. PCOS-IR patients (*p*<0.0005; Table 3). A total of 40 genes were identified as differentially methylated in PCOS vs. healthy controls (*p*<0.0005; Table 4).

**Table 3:** The 79 differentially methylated genes in PCOS-NIR patients vs. PCOS-IR patients (p<0.0005).

Gene ID	Official Symbol	P value	Gene ID	Official Symbol	P value
**398**	ARHGDIG	0.00036	84307	ZNF397	0.00035
**83864**	TTTY9A	0.00019	4606	MYBPC2	0.00004
**55013**	CCDC109B	0.00019	90427	BMF	0.00028
**283464**	GXYLT1	0.00006	1636	ACE	0.00037
**494514**	C18orf56	0.00039	341457	PPIAP8	0.00027
**60673**	C12orf44	0.00011	8536	CAMK1	0.00038
**127829**	ARL8A	0.00003	84896	ATAD1	0.00015
**51608**	GET4	0.00031	3978	LIG1	0.00002
**8493**	PPM1D	0.00027	91662	NLRP12	0.00015
**358**	AQP1	0.00030	5028	P2RY1	0.00030
**1120**	CHKB	0.00043	1119	CHKA	0.00040
**433**	ASGR2	0.00047	440107	PLEKHG7	0.00037
**3266**	ERAS	0.00016	284996	RNF149	0.00024
**340120**	ANKRD34B	0.00023	2618	GART	0.00048
**6013**	RLN1	0.00006	5480	PPIC	0.00021
**153328**	SLC25A48	0.00044	7087	ICAM5	0.00040
**8320**	EOMES	0.00005	285679	C5orf60	0.00022
**3241**	HPCAL1	0.00006	51465	UBE2J1	0.00031
**51529**	ANAPC11	0.00048	145942	TMCO5A	0.00032
**6340**	SCNN1G	0.00037	9240	PNMA1	0.00020
**389941**	C1QL3	0.00030	25894	PLEKHG4	0.00047
**2805**	GOT1	0.00030	164091	PAQR7	0.00030
**128322**	LOC128322	0.00036	7296	TXNRD1	0.00040
**53820**	DSCR6	0.00025	9659	PDE4DIP	0.00049
**6531**	SLC6A3	0.00045	79854	LINC00115	0.00007
**84698**	CAPS2	0.00018	165530	CLEC4F	0.00025
**85027**	SMIM3	0.00023	644613	LOC644613	0.00012
**9525**	VPS4B	0.00003	8439	NSMAF	0.00027
**645369**	TMEM200C	0.00017	4255	MGMT	0.00026
**64417**	C5orf28	0.00001	1051	CEBPB	0.00017
**55333**	SYNJ2BP	0.00045	115548	FCHO2	0.00028
**4953**	ODC1	0.00020	55020	TTC38	0.00011
**644765**	LOC644765	0.00041	51646	YPEL5	0.00050
**10799**	RPP40	0.00047	57684	ZBTB26	0.00038
**389384**	C6orf222	0.00048	111	ADCY5	0.00028
**80724**	ACAD10	0.00038	4065	LY75	0.00018
**83858**	ATAD3B	0.00031	2837	UTS2R	0.00010
**195828**	ZNF367	0.00013	254312	LINC00710	0.00004
**56957**	OTUD7B	0.00033	163049	ZNF791	0.00021
**1746**	DLX2	0.00024			

**Table 4:** The 40 differentially methylated genes in PCOS vs. healthy controls (p<0.0005).

Gene ID	Official Symbol	P value	Gene ID	Official Symbol	P value
**120425**	AMICA1	0.00001	164781	WDR69	0.00022
**166348**	KBTBD12	0.00002	245929	DEFB115	0.00023
**2030**	SLC29A1	0.00003	54826	GIN1	0.00026
**11245**	GPR176	0.00005	158038	LINGO2	0.00027
**51778**	MYOZ2	0.00005	390084	OR56A5	0.00028
**51604**	PIGT	0.00005	158405	KIAA1958	0.00028
**388125**	C2CD4B	0.00005	643812	KRTAP27-1	0.00030
**56141**	PCDHA7	0.00006	59269	HIVEP3	0.00031
**3159**	HMGA1	0.00008	9532	BAG2	0.00033
**54510**	PCDH18	0.00010	56674	TMEM9B	0.00034
**6738**	TROVE2	0.00010	221914	GPC2	0.00037
**374928**	ZNF773	0.00010	2324	FLT4	0.00039
**6336**	SCN10A	0.00011	644624	LOC644624	0.00039
**220766**	CEP170L	0.00011	64928	MRPL14	0.00040
**375287**	RBM43	0.00012	445582	POTEE	0.00040
**53838**	C11orf24	0.00015	1984	EIF5A	0.00044
**6319**	SCD	0.00015	1047	CLGN	0.00045
**128488**	WFDC12	0.00020	4062	LY6H	0.00047
**3676**	ITGA4	0.00021	441268	LOC441268	0.00048
**2931**	GSK3A	0.00022	10156	RASA4	0.00048

### Construction of regulatory network

**Figure 2:**
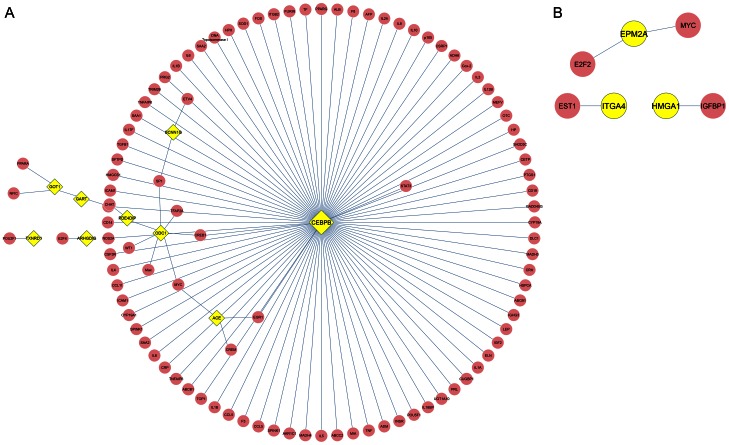
Regulatory network analysis of differentially methylated genes. A. regulatory network of differentially methylated genes between PCOS-NIR and PCOS-IR patients; B. regulatory network of differentially methylated genes between PCOS patients and healthy controls. The yellow nodes represent methylated genes and pink nodes represent normal genes.

To get the regulatory relationships between PCOS-NIR and PCOS-IR patients as well as between PCOS patients and healthy control, we mapped their differentially methylated genes into regulation data collected from TRANSFAC and TRED, and built regulatory networks by Cytoscape software [24] (Figure 2A and B). In the regulatory network between PCOS-NIR and PCOS-IR, significant difference in CEBPB gene methylation was observed (*p* = 0.00017). CEBPB formed a local network by regulating a number of genes, suggesting it may play an important role in PCOS-IR. Besides, CEBPB indirectly regulated the methylated gene ODC1 through regulating the normal gene (unmethylated gene) CREB1. In our network, we observed that methylated gene GART regulated the methylated gene GOT1 directly, and regulated another methylated gene PDE4DIP indirectly (Figure 2A). The regulatory network of differentially methylated genes between PCOS patients and healthy controls was much simpler. In this network, the methylated gene EPM2A regulated two normal genes, MYC and E2F2. The methylated genes ITGA4 and HMGA1 regulated the normal genes ETS1 and IGFBP1, respectively (Figure 2B).

### Gene Ontology (GO) function analysis of regulatory network

To explore the biological function of genes in the regulatory network of PCOS-NIR vs. PCOS-IR, we applied the online biological classification tool DAVID and observed significant enrichments of these differentially methylated genes in multiple GO categories (Table 5). The most significant enrichment was GO category of defense response with FDR = 3.16E-18. The other significant GO categories included inflammatory response (FDR = 1.73E-17), response to wounding (FDR = 9.91E-17) and regulation of cytokine production (FDR = 2.09E-12). In fact, all significant GO category clusters were associated with immune response (Table 5).

**Table 5:** GO function analysis of regulatory network of PCOS-NIR vs. PCOS-IR.

Term	Description	Count	FDR
**GO:0006952**	defense response	33	3.16E-18
**GO:0006954**	inflammatory response	26	1.73E-17
**GO:0009611**	response to wounding	30	9.91E-17
**GO:0001817**	regulation of cytokine production	18	2.09E-12
**GO:0006953**	acute-phase response	11	7.78E-11
**GO:0031328**	positive regulation of cellular biosynthetic process	27	9.00E-11
**GO:0009891**	positive regulation of biosynthetic process	27	1.26E-10
**GO:0010557**	positive regulation of macromolecule biosynthetic process	26	2.65E-10
**GO:0051240**	positive regulation of multicellular organismal process	18	2.91E-10
**GO:0010033**	response to organic substance	27	2.97E-10

### Pathway analysis of regulatory network

To identify the deregulated pathways in patients with PCOS-IR vs. PCOS-NIR, we performed pathway enrichment analysis on the differentially methylated genes using the online tool of DAVID (Table 6). At a FDR of 0.01, four pathways were enriched, including cytokine-cytokine receptor interaction (FDR = 8.39E-06), hematopoietic cell lineage (FDR = 2.76E-04), asthma (FDR = 5.24E-04) and Jak-STAT signaling pathway (FDR = 6.15E-04) (Table 6).

**Table 6:** Pathway analysis of regulatory network of PCOS-NIR vs. PCOS-IR.

Term	Description	Count	FDR
**hsa04060**	Cytokine-cytokine receptor interaction	17	8.39E-06
**hsa04640**	Hematopoietic cell lineage	10	2.76E-04
**hsa05310**	Asthma	7	5.24E-04
**hsa04630**	Jak-STAT signaling pathway	12	6.15E-04

### Construction of protein-protein interaction (PPI) networks

**Figure 3:**
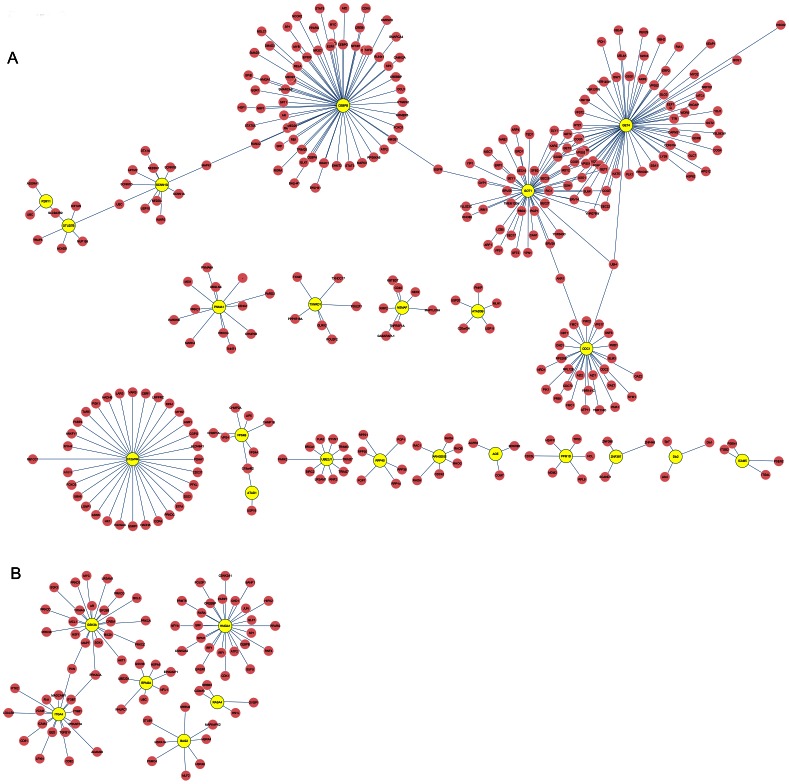
Protein-protein interaction (PPI) network analysis. A. Protein-protein interaction (PPI) network of PCOS-NIR and PCOS-IR; B. PPI network of PCOS and controls. Yellow nodes represent methylated genes and pink nodes represent normal genes.

Transcriptional changes are not always strictly correlated with protein expressions and functions. To investigate the differentially methylated genes in protein level, we constructed PPI networks between PCOS-NIR and PCOS-IR as well as PCOS and healthy controls through analyzing the data collected from HPRD and BIOGRID (Figure 3). The importance of a gene is often dependent on how well it associates with other genes in a network. Studies suggest that more centralized genes in the network are more likely to be key drivers to proper cellular function than peripheral genes (nodes) [25]. From the PPI network of PCOS-NIR vs. PCOS-IR, we observed that the methylated genes CEBPB, GOT1, GET4, ODC1 and C12orf44 formed local networks (Figure 3A). In the PPI network of PCOS vs. healthy controls, the methylated genes GSK3A, HMGA1, ITGA4, EPM2A and BAG2 were hub nodes (Figure 3B).

### GO function analysis of PPI network

To investigate the biological function of genes in PPI networks, we performed GO function analysis for these genes in each PPI network, respectively. Table 7 shows the top 10 enriched GO gene categories in the PPI network of PCOS-NIR vs. PCOS-IR. The most significant GO gene category was regulation of transcription of RNA polymerase II promoter (FDR = 8.38E-22). The other significant GO categories included positive regulation of nitrogen compound metabolic process (FDR = 1.21E-19), positive regulation of macromolecule metabolic process (FDR = 4.40E-18) and positive regulation of macromolecule metabolic process (FDR = 6.68E-18) (Table 7). Table 8 shows the top 10 GO categories in the PPI network of PCOS vs. healthy controls. The most significant GO category was positive regulation of macromolecule metabolic process (FDR = 5.47E-11). The other significant GO categories included positive regulation of cellular biosynthetic process (FDR = 2.43E-06), positive regulation of biosynthetic process (FDR = 3.15E-06) and positive regulation of nitrogen compound metabolic process (FDR = 5.17E-06) (Table 8).

**Table 7:** GO function analysis of PPI network of PCOS-NIR vs. PCOS-IR.

Term	Description	Count	FDR
**GO:0006357**	regulation of transcription from RNA polymerase II promoter	62	8.38E-22
**GO:0051173**	positive regulation of nitrogen compound metabolic process	56	1.21E-19
**GO:0010604**	positive regulation of macromolecule metabolic process	62	4.40E-18
**GO:0045935**	positive regulation of nucleobase, nucleoside, nucleotide and nucleic acid metabolic process	53	6.68E-18
**GO:0010628**	positive regulation of gene expression	49	4.02E-16
**GO:0045941**	positive regulation of transcription	48	7.12E-16
**GO:0045893**	positive regulation of transcription, DNA-dependent	44	1.50E-15
**GO:0051254**	positive regulation of RNA metabolic process	44	2.06E-15
**GO:0031328**	positive regulation of cellular biosynthetic process	52	2.26E-15
**GO:0045944**	positive regulation of transcription from RNA polymerase II promoter	39	2.68E-15

**Table 8:** GO function analysis of PPI network of PCOS vs. healthy controls.

Term	Description	Count	FDR
**GO:0010604**	positive regulation of macromolecule metabolic process	30	5.47E-11
**GO:0031328**	positive regulation of cellular biosynthetic process	22	2.43E-06
**GO:0009891**	positive regulation of biosynthetic process	22	3.15E-06
**GO:0051173**	positive regulation of nitrogen compound metabolic process	21	5.17E-06
**GO:0010628**	positive regulation of gene expression	20	5.93E-06
**GO:0010557**	positive regulation of macromolecule biosynthetic process	21	6.73E-06
**GO:0006468**	protein amino acid phosphorylation	21	9.42E-06
**GO:0045893**	positive regulation of transcription, DNA-dependent	18	1.26E-05
**GO:0051254**	positive regulation of RNA metabolic process	18	1.43E-05
**GO:0045935**	positive regulation of nucleobase, nucleoside, nucleotide and nucleic acid metabolic process	20	1.90E-05

### Pathway analysis of PPI network

Furthermore, we performed the pathway enrichment analysis for these genes in PPI network. Table 9 shows pathways enriched in PPI network of PCOS-NIR vs. PCOS-IR. At a FDR of 0.01, 6 pathways were enriched, including pathways in cancer (FDR = 4.78E-08), chronic myeloid leukemia (FDR = 3.17E-05), and prostate cancer (FDR = 2.62E-04) (Table 9). Table 10 showed the enriched pathways in PPI network of PCOS vs. healthy controls. At a FDR of 0.01, 5 pathways were enriched, including pathways in cancer (FDR = 0.00112), ErbB signaling pathway (FDR = 0.001209), and focal adhesion (FDR = 0.001848) (Table 10).

**Table 9:** Pathway analysis of PPI network of PCOS-NIR vs. PCOS-IR.

Term	Description	Count	FDR
**hsa05200**	Pathways in cancer	32	4.78E-08
**hsa05220**	Chronic myeloid leukemia	14	3.17E-05
**hsa05215**	Prostate cancer	14	2.62E-04
**hsa05221**	Acute myeloid leukemia	11	0.001466
**hsa05212**	Pancreatic cancer	12	0.001485
**hsa05219**	Bladder cancer	9	0.008231

**Table 10:** Pathway analysis of PPI network of PCOS vs. healthy controls.

Term	Description	Count	FDR
**hsa05200**	Pathways in cancer	15	0.00112
**hsa04012**	ErbB signaling pathway	9	0.001209
**hsa04510**	Focal adhesion	12	0.001848
**hsa04010**	MAPK signaling pathway	13	0.00462
**hsa04310**	Wnt signaling pathway	10	0.009299

## Discussion

PCOS affects 6–10% of women of childbearing age, many groups suggested that insulin resistance plays a critical role in PCOS development [26], [27]. Despite significant research advances have been achieved over the past decade [28], many questions remain uncertain. In the current study, we employed genome-wide methylated DNA immunoprecipitation (MeDIP) analysis to characterize genes that are differently methylated between PCOS patients and healthy controls, or between PCOS-NIR vs. PCOS-IR patients. Besides, we constructed the regulatory networks and PPI networks after analyzing these differentially methylated genes in PCOS-NIR vs. PCOS-IR, or in PCOS vs. control. Furthermore, the GO function and pathway analysis were performed for regulatory networks and PPI networks. We found various GO categories were enriched including cytokine-cytokine receptor interaction, hematopoietic cell lineage, and asthma. Bio-pathway analysis for these genes in PPI network showed that cancer pathways were enriched after comparing PCOS-NIR with PCOS-IR patients, as well as comparing PCOS patients with healthy controls.

DNA methylation is an epigenetic modification associated with gene transcription regulation, X-chromosome inactivation, development and cell differentiation regulation. Aberrant DNA methylation is closely associated with cancer development and progression. The advent of microarray technology has provided new opportunities for high-throughput study on DNA methylation. Microarray-based methods include immunoprecipitation and restriction digestion. Each technique has its own advantages. Immunoprecipitation uses the specificity of antibodies to isolate target proteins (antigens) out of complex sample mixtures [29]. Restriction enzyme digestion using methylcytosine-sensitive enzymes, followed by ligation-mediated PCR amplification of the targets [30]. Therefore, the immunoprecipitation method is more specific while the restriction digestion method is more sensitive. Together, they provide many choices for the study of genome-wide DNA methylation profile in disease.

In order to further confirm the specificity of methylation, we performed a MSP using estrogen receptor beta (ER-βER-β) gene. ER-βER-β is expressed by many tissues and its expression can be regulated by DNA methylation of the promoter region. Previous study suggested that the methylation of ER-βER-β is related to genesis of tumor and endocrine disease [31]. Besides, the ER-βER-β gene polymorphism was reported to be associated with pathophysiologic aberrancies involved in PCOS [32]. From Figure 1, we could find that the fragment of 250 bp appeared in samples amplified by M primer. The fuzzy band in the U lane indicated partially methylated DNA fragment.

From Table 2, we could find that significant difference in CEBPB gene methylation was observed between PCOS-NIR patients and PCOS-IR patients (*p* = 0.000170). Besides, CEBPB formed local networks in both regulatory network (Figure 2A) and PPI network (Figure 3A). These results all suggested CEBPB plays an important role in insulin resistance in PCOS patients. CEBPB is a bZIP transcription factor which can bind as a homodimer to certain DNA regulatory regions. CEBPB is important in the regulation of genes involved in immune and inflammatory responses and has been shown to bind to the interleukin (IL) −1 response element in the IL- 6 gene, as well as to regulatory regions of several acute- phase and cytokine genes [33]. Expression of CEBPB in blood leukocytes has been shown to be positively associated with muscle strength in humans, emphasizing the importance of the immune system [34]. In particular, CEBPB is a downstream effector of the luteinizing hormone signaling pathway and thus plays key roles in the luteinizing hormone response of the follicle [35]. CEBPB is involved in the acquisition of insulin receptor substrate (IRS) −2 and glucose transporter 4 (GLUT4) expression as well as in insulin - sensitive glucose uptake during adipocyte differentiation [36]. We could find a significant difference of CEBPB gene methylation between PCOS-NIR and PCOS-IR patients (*p* = 0.00017), suggesting CEBPB involving in insulin resistance in PCOS patients. Besides, CEBPB indirectly regulated the methylated gene ODC1 through regulating the normal gene CREB1, as shown in Figure 2A. ODC1 (ornithine decarboxylase 1) is a rate-limiting enzyme of the polyamine biosynthesis pathway which catalyzes ornithine to putrescine. A previous study suggested that exposure to ethanol results in insulin resistance and thereby disrupts the molecular path by which induces the expression of ODC enzymatic activity [37], indicating the role of ODC1 in insulin resistance.

As shown in Table 5, genes of defense response, inflammatory response, and the response to wounding belong to the cellular immunity term were differentially methylated in PCOS vs healthy controls, suggesting that PCOS may be associated with the immune response. The immune response is how your body recognizes and defends itself against bacteria, viruses, and substances that appear foreign and harmful [38]. An efficient immune response protects against many diseases and disorders. The gene categories of regulation of transcription from RNA polymerase II promoter, positive regulation of macromolecule metabolic process, positive regulation of transcription, DNA-dependent and positive regulation of cellular biosynthetic process appeared in both GO function and pathway analysis. These genes are all necessary in biological growth and differentiation, proliferation and development [39]. The biosynthesis process often consists of several enzymatic steps in which the product of one step is used as substrate in the following step. Examples for such multi-step biosynthetic pathways are those for the production of amino acids, fatty acids, and natural products [40]. Biosynthesis plays a major role in all cells, and many dedicated metabolic routes combined constitute general metabolism. Both PCOS-NIR and PCOS-IR were related to biosynthesis.

Table 9 and Table 10 showed that the category of genes related to pathways in cancer were differently methylated PCOS-NIR and PCOS-IR. The abnormal activation of signaling pathways is a critical event in cancer pathogenesis [41]. In particular, activation of these pathways can lead to inappropriate cellular survival, proliferation, pluripotency, invasion, metastasis, and angiogenesis [41].
